# Changes in corneal densitometry after long-term orthokeratology for myopia and short-term discontinuation

**DOI:** 10.1371/journal.pone.0263121

**Published:** 2022-02-04

**Authors:** Lianghui Zhao, Lili Jing, Jie Li, Xianli Du

**Affiliations:** 1 Qingdao Eye Hospital of Shandong First Medical University, Qingdao, China; 2 State Key Laboratory Cultivation Base, Shandong Provincial Key Laboratory of Ophthalmology, Shandong Eye Institute, Shandong First Medical University & Shandong Academy of Medical Sciences, Qingdao, China; 3 Weifang Medical University, Weifang, China; Saarland University, GERMANY

## Abstract

**Purpose:**

To quantify changes in corneal densitometry after long-term orthokeratology treatment in myopic children and to analyze the reversibility one month after discontinuation.

**Methods:**

Seventy-four myopic subjects aged 8–16 years, who wore orthokeratology lenses for two years, were divided into relatively steep- (lens movement within 1.0–1.5 mm, thirty-six participants) and flat-fitting groups (lens movement within 1.5–2.0 mm, thirty-eight participants). Based on refractive errors, they were divided into low and moderate myopia groups (thirty-seven participants in each group). Corneal densitometry was performed using Pentacam (Oculus Optikgeräte GmbH, Wetzlar, Germany) at each follow-up timepoint. Repeated-measures analysis of variance was used to compare the parameters before and after orthokeratology.

**Results:**

The corneal densitometry values over the 0–10 mm diameter area increased from 12.84±1.38 grayscale units (GSU) at baseline to 13.59±1.42 GSU after three-month orthokeratology (*P* = .001) and reached 14.92±1.45 GSU at two years (*P* < .001). An increase in densitometry began at one month (*P* = .001) over the 0–2 mm annulus compared with that at three months over the 2–6 mm and 6–10 mm zones (*P* = .002,.014). The densitometry values significantly increased at three months in the relatively steep-fitting group (*P* = .003) and at one year in the relatively flat-fitting group (*P* = .001). After discontinuation of orthokeratology for one month, the values showed no significant decrease.

**Conclusions:**

Long-term orthokeratology treatment causes a small but statistically significant increase in corneal densitometry values. During the first year, the onset of these changes was related to the fitting mode. Corneal densitometry values showed no significant reduction after one-month discontinuation.

## Introduction

As orthokeratology has become a valid method to control myopia and has been used worldwide in recent years [1, 2], the safety of the cornea associated with the technique has increasingly become a concern. Previous studies have found that a brownish pigmented corneal arc can appear after orthokeratology [3–5], which may change the transparency of some areas of the cornea. Therefore, we speculate that corneal transparency might change after long-term orthokeratology treatment, but the change is too slight to be quantified by slit-lamp microscopy.

Corneal transparency, an important safety index of the cornea, depends not only on the normal functions of corneal epithelial cells, stromal cells, and endothelial cells but also on the regular arrangement of fibers. Orthokeratology lenses can alter the epithelial thickness profile with central thinning and mid-peripheral thickening [6–8]. Histological evidence has shown that these changes are associated with the compression of central basal cells, the elongation of mid-peripheral basal cells, and the increased number of mid-peripheral epithelial cell layers after orthokeratology treatment [9]. Moreover, the density and morphology of corneal stromal cells have been shown to change after orthokeratology lens wear [10].

Corneal transparency can be evaluated quantitatively using the Pentacam software, and corneal densitometry can be used as the quantitative index. This non-invasive optical examination method has been extensively used in diagnosing eye diseases and evaluating corneal surgery [11–16]. Changes of corneal densitometry have been assessed after long-term of soft contact lenses wearing [17] and also been detected after one year of orthokeratology treatment [18], but there have been no reports about the outcomes of corneal densitometry after long-term orthokeratology or discontinuation of lens wear. In this study, we sought to describe the changes in corneal densitometry over the 0–10 mm diameter area during two-year orthokeratology treatment in myopic children, to analyze the reversibility of these changes after short-term discontinuation following treatment, and to evaluate the possible influencing factors.

## Participants and methods

### Participants

This Retrospective cohort study was approved by the institutional review board of Qingdao Eye Hospital and was carried out in accordance with the tenets of the Declaration of Helsinki. Medical records of participants who had orthokeratology treatment at the Qingdao Eye Hospital between January 2014 and December 2018 were reviewed. Seventy-four children (thirty-six males and thirty-eight females) aged 8–16 years (10.43 ± 2.03 years) who wore orthokeratology lenses for the first time and continued this treatment for at least two years were included in this study. Signed informed consent was obtained from their guardians. Of the participants, fifty-four discontinued lenses wearing for more than four weeks after two years of orthokeratology treatment.

The inclusion criteria were as follows: best-corrected visual acuity higher than or equal to 20/20, spherical refractive errors less than or equal to 6.00 diopters, and astigmatism less than or equal to 1.50 diopters. All eyes had a clear cornea, with no history of ocular disease, trauma, or surgery. All orthokeratology lenses were centered on the cornea with good mobility and no obvious deviation. No inflammation occurred during the treatment period. One eye was randomly chosen from each patient for evaluation by coin toss; if only one eye in a patient received orthokeratology, the treated eye was involved.

All the participants were fitted with orthokeratology lenses and were recommended to wear them for at least eight consecutive hours every night. To assess the impact of the fitting modes on corneal densitometry, the children were grouped based on the lens movement and the fluorescein pattern, with the fitting mode tolerated by each participant. In the relatively steep-fitting group (thirty-eight participants), the lens movement was controlled within 1.0–1.5 mm, and the fluorescein area of the optic zone was smaller than the normal. In addition, the fluorescein pooling in the alignment zone was dark green, with a few tears under the lens. The peripheral zone was narrower compared to the normal. There were occasionally some bubbles under the reverse zone or optic zone. In the relatively flat-fitting group (thirty-six participants), the movement of the lens was about 1.5–2.0 mm. The fluorescein area of the optic zone was larger than the normal, and the fluorescein pooling in the alignment zone was bright green, with more tears under the lens. The peripheral zone was wider compared to the normal. To evaluate the correlation between refractive errors and corneal densitometry, the participants were also divided into a low myopia group (-1.00 to -3.00 D, 37 participants) and a moderate myopia group (-3.25 to -6.00 D, 37 participants).

### Lenses

All orthokeratology lenses were made of Boston XO material (Polymer Technology Corporation, Boston, MA, USA). The lens parameters provided by the manufacturer were as follows: total diameter was 10.00 to 11.50 mm; base curve radius was 7.50 to 9.93 mm; visible light transmittance was higher than 88%; refractive index was 1.415; center thickness was 0.15 to 0.30 mm; oxygen permeability (Dk) was 100×10^−11^ (cm^2^/s) (ml O_2_/ml·mmHg); oxygen transmissibility (Dk/t) at the center thickness (0.18 mm) at -3.00 D was 55.56×10^−9^ (cm/s) (ml O_2_/ml·mmHg).

### Examinations

Slit-lamp microscopy and corneal tomography were performed before orthokeratology treatment; after one, three, six, twelve, and twenty-four months of lens wearing; and after discontinuing the two-year treatment for one month. Examinations were all conducted in the afternoon, 6–8 h after lens removal. To ensure the accuracy and reliability of the examination results, the Pentacam was calibrated and monitored with a standard test chip by senior technicians from the manufacturer every three months.

Corneal densitometry values were collected during corneal tomography examinations and were analyzed using the standardized Scheimpflug densitometry analysis add-on to the standard software of the Pentacam Scheimpflug device (Oculus Optikgeräate GmbH, Wetzlar, Germany). The Pentacam system was used to determine the light scatter in the defined zones by grayscale units (GSUs), with a maximum transparency of 0 GSU and a minimum transparency of 100 GSU. The GSU scale was calibrated by proprietary software, and the Pentacam scans with a quality specification of “OK” were chosen for analysis. Three high-quality images were taken of each patient, and the mean value of the three results was used for final statistical analysis. Corneal densitometry was automatically measured over a 12-mm diameter area in the full-thickness cornea, which was divided into annular concentric zones of 0–2 mm, 2–6 mm, 6–10 mm, and 10–12 mm. The data of the peripheral 10–12 mm was excluded from the analysis, as this zone is already known to have a lowest degree of repeatability (6.4%) and a lowest reproducibility (3.1%) [19, 20].

### Statistical analysis

Repeatability was assessed by within-subject standard deviation (Sw) and coefficient of variation (CVw). Statistical analyses were performed using Microsoft Excel (version 2019; Microsoft, Redmond, WA, USA) and Statistical Package for Social Sciences software (version 24.0). All the data were first examined with the Kolmogorov–Smirnov test to evaluate the normality and these data were normally distributed. The descriptive statistics were presented as the mean ± standard deviation. Repeated-measures analysis of variance was used to compare the parameters before and after orthokeratology. The equality of variances and sphericity were tested using Levene and Mauchly tests. Least significant difference tests were used to determine the differences between pairs of comparisons. The significance level for all the tests was set to 5%.

## Results

The range of within-subject standard deviation and coefficient of variation before and after orthokeratology treatment in the 0–10 mm diameter area (0.5%–0.8% and 4%–7%; 0.8%–0.9% and 5%–7%) was significantly less variable than that in the 10–12 mm area (5.9% and 29%; 3.9% and 19%) (Table 1).

**Table 1:** Within-subject standard deviation (Sw) and coefficient of variation (CVw) of densitometry measurements (95% confidence intervals of the estimates are shown in parentheses).

Area	Before lens wearing		twelve months after lens wearing	
	Sw	CVw	Sw	CVw
0–2 mm	0.5 (0.5–0.6)	0.04 (0.036–0.042)	0.8 (0.8–0.9)	0.05 (0.053–0.057)
2–6 mm	0.5 (0.4–0.5)	0.04 (0.042–0.045)	0.8 (0.8–0.9)	0.06 (0.059–0.061)
6–10 mm	0.8 (0.8–0.9)	0.07 (0.066–0.074)	0.9 (0.9–1.0)	0.07 (0.069–0.077)
10–12 mm	5.9 (6.1–6.2)	0.29 (0.282–0.300)	3.9 (4.0–4.1)	0.19 (0.181–0.193)

**Fig 1:**
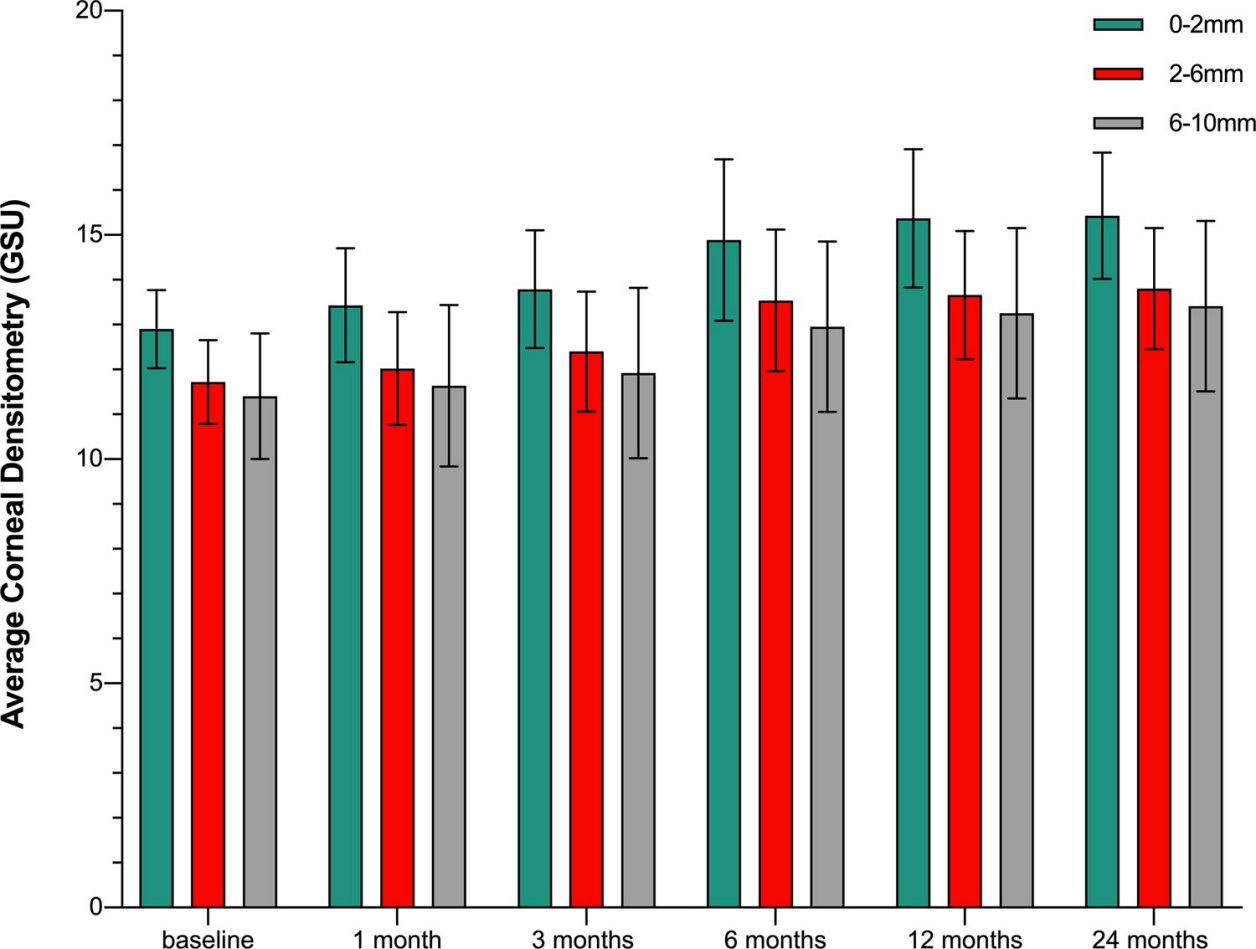
Changes in the corneal densitometry values at different time points with various corneal diameters.

No significant change was found in the corneal densitometry values early after orthokeratology. The baseline densitometry value over the 0–10 mm diameter area was 12.84±1.38 GSU, and it increased significantly to 13.59 ± 1.42 GSU at three months (*P* = .001) and reached 14.92 ± 1.45 GSU at two years (*P* < .001) (Table 2). The corneal densitometry over the 0–2 mm diameter area began to increase significantly at one month, whereas that over the 2–6 mm and 6–10 mm zones began to increase significantly at three months (*P* = .001, .002, .014). The values of these three diameter areas increased over time (*P* < .001) (Table 3 and Fig 1).

**Table 2:** Changes in the average corneal densitometry values over the 0–10 mm diameter area between baseline and post-treatment (represented in grayscale units, GSU).

Time	Average corneal densitometry (GSU)	P
Baseline	12.84±1.38	
one month follow-up	13.15±1.49	.058
three months follow-up	13.59±1.42	.001
six months follow-up	14.36±1.89	< .001
twelve months follow-up	14.78±1.68	< .001
twenty-four months follow-up	14.92±1.45	< .001

Data are provided as mean ± standard deviation. The value of P is the comparison of each time point and baseline. There were statistically significant differences over time (F = 26.891, P<0.001).

**Table 3:** Changes in the corneal densitometry values in seventy-four patients over different annular concentric zones between baseline and post-treatment (represented in grayscale units, GSU).

Time	0–2 mm (GSU)	P	2–6 mm (GSU)	P	6–10 mm (GSU)	P
Baseline	12.90±0.87		11.72±0.93		11.39±1.84	
one month follow-up	13.43±1.27	.001	12.02±1.26	.058	11.64±1.89	.107
three months follow-up	13.81±1.31	< .001	12.40±1.34	.002	11.92±1.90	.014
six months follow-up	14.87±1.80	< .001	13.54±1.58	< .001	12.95±1.97	< .001
twelve months follow-up	15.36±1.54	< .001	13.66±1.43	< .001	13.25±1.97	< .001
twenty-four months follow-up	15.43±1.41	< .001	13.80±1.35	< .001	13.41±1.93	< .001

Data provided as mean ± standard deviation. The value of P is the comparison of each time point and baseline. There were statistically significant differences over time (0–2 mm: F = 34.754, P < .001; 2–6 mm: F = 27.995, P < .001; 6–10 mm: F = 21.123, P < .001).

No significant difference was found in the corneal densitometry values between the low myopia group and the moderate myopia group (*F* = 27.465, *P* >.05). In other words, the corrected refractive error had no effect on the changes in corneal densitometry after orthokeratology. After one month’s discontinuation, the corneal tomography showed that the deformation of the cornea by orthokeratology was disappeared, and the patient’s corneal morphology returned to the preoperative level. And the densitometry values over different areas showed an insignificantly decreasing trend at one month after the discontinuation of lens wear compared with those at twenty-four months after lens wear (P >.05), but they increased significantly compared with those before treatment (Table 4).

**Table 4:** Changes in the corneal densitometry values in fifty-four patients over different annular concentric zones between baseline and post-treatment (represented in grayscale units, GSU).

Time	0–2 mm (GSU)	P	2–6 mm (GSU)	P	6–10 mm (GSU)	P
Baseline	13.18±1.12		12.00±1.08		11.31±1.41	
twenty-four months follow-up	15.24±1.28	< .001	13.77±1.51	< .001	12.96±1.92	< .001
one month after discontinuation	14.93±1.07	< .001	13.44±1.07	< .001	12.79±1.36	< .001

Data provided as mean ± standard deviation. The value of P is the comparison of each time point and baseline. There were statistically significant differences over time (0–2 mm: F = 50.38, P < .001; 2–6 mm: F = 37.718, P < .001; 6–10 mm: F = 29.502, P < .001).

**Fig 2:**
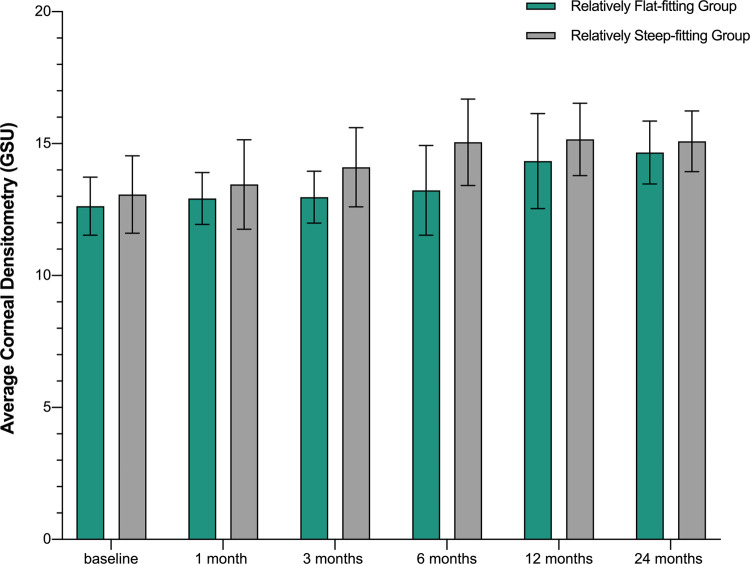
Changes in the mean corneal densitometry values in eyes with various fitting modes at different time points.

The densitometry values in the relatively steep-fitting group increased earlier than those in the relatively flat-fitting group (at three months vs. at one year; *P* = .003, .001). At two years, the fitting methods were no longer the main influencing factor of corneal densitometry, and there was no significant correlation between them (Table 5, Fig 2).

**Table 5:** Changes in the corneal densitometry values in eyes with different fitting modes between baseline and post-treatment (represented in grayscale units, GSU).

Time	relatively flat-fitting group		relatively steep-fitting group	
	densitometry (GSU)	P	densitometry (GSU)	P
Baseline	12.63±1.19		13.07±1.47	
one month follow-up	12.92±0.98	.173	13.45±1.79	.158
three months follow-up	12.97±0.98	.118	14.10±1.56	.003
six months follow-up	13.23±1.75	.185	15.05±1.64	< .001
twelve months follow-up	14.34±1.84	.001	15.16±1.37	< .001
twenty-four months follow-up	14.66±1.19	< .001	15.09±1.15	< .001

Data provided as mean ± standard deviation. The value of P is the comparison of each time point and baseline. There were statistically significant differences over time (F = 31.266, P < .001).

## Discussion

Orthokeratology may cause corneal pigmented arcs, and the changes in corneal transparency can be observed by slit-lamp microscopy but cannot be quantified due to the limitations of examination methods. Previous studies found that and the loss of corneal transparency may cause increased corneal light scattering and thus straylight. Straylight measurements can reflect the early subclinical changes of corneal microstructure, which is more sensitive than vision or slit-lamp, and rigid contact lens can lead to increased straylight [21]. Nowadays, with the advent of standardized Scheimpflug densitometry analysis software, corneal densitometry as a quantitative indicator of transparency has been used in the investigations on keratoconus [11], Fuchs endothelial dystrophy [12], refractive surgery [13], corneal collagen crosslinking [14], Descemet membrane endothelial keratoplasty [15] and quantitative evaluation of corneal epithelial edema after cataract surgery [16]. However, little is known about the changes in corneal densitometry related to orthokeratology [18]. In this study, corneal densitometry was measured before and after long-term orthokeratology to reflect the minimal subclinical changes in corneal transparency.

In our series, we observed corneal densitometry during the two-year orthokeratology treatment and found that the corneal densitometry values increased about 2 GSU. As Koc reported, the densitometry in the anterior cornea of the 0–2 mm zone of keratoconus patients increased almost 5 GSU compared to controls, which was associated with early epithelial structural changes and stroma structural degeneration [20]. Ishikawa et al. found an approximately 3 GSU increase one day after cataract surgery, which implied the occurrence of subclinical corneal epithelial or stromal edema [16]. Ding et al. reported that after six months of orthokeratology treatment, densitometry increased about 2 GSU and corneal transparency decreased [18]. Compared with previous studies, orthokeratology might lead to minimal changes in corneal transparency, and this subclinical change can be quantified by densitometry.

By assessing corneal densitometry values over different corneal annulus zones, we found an initial increase in corneal densitometry over the 0–2 mm diameter area at the timepoint of one month. This was consistent with the finding of Nieto-Bona et al. that the activation of stromal cells started after one month of corneal shaping [22]. We also demonstrated that the densitometry values over the 2–6 mm and 6–10 mm areas began to increase at three months. The reasons for the changes may be as follows. The 0–2 mm annulus was one of the first contacting areas between the orthokeratology lens and the cornea; the mechanical stimulation-induced cellular activation occurred first in this zone, leading to changes in the corneal epithelial cells and the remodeling of corneal stroma. The 2–6 mm area was located at the periphery of the optical region, where the accumulation of corneal shaping force could play a role in changes to corneal densitometry. The 6–10 mm area roughly corresponded with the reverse curve and alignment curve area of the orthokeratology lens; it could not be subdivided effectively by the analysis software and was thus analyzed as a unit. We speculated that the tear accumulation and the effect of mechanical compression might be related to the corneal transparency in this unit.

In this study, the fitting methods were found to affect changes in corneal densitometry during the first year of lens wearing. The corneal densitometry values in the relatively steep-fitting group were shown to be significantly increased after three months’ lens wear, earlier than those in the relatively flat-fitting group (one year). However, the two fitting modes produced roughly the same results after twenty-four months. At present, the mechanism of this interesting phenomenon is not clear, and further basic research is still needed.

Regarding the reversibility of changes in corneal tissue after lens discontinuation, previous studies have shown that changes could be reversed within a short period [4, 22–27]. The morphology of the cells in the stromal layers (increased keratocyte activation) and epithelial layers (decreased cell densities and increased cell areas) in response to orthokeratology treatment may return to baseline after discontinuing treatment [22]. We also assessed the recovery of corneal densitometry, finding that the corneal densitometry values did not return to baseline after discontinuation of lens wear. This may be attributed to the long duration of orthokeratology and subsequent short discontinuation. The pigmented arc and the white lesion were reported to regress after 6 months of lens replacement [27]. During our two-year follow-up, it took three months to observe obvious changes after orthokeratology, whereas the treatment was discontinued for only one month. Therefore, further studies with a longer follow-up time after discontinuing treatment are needed to clarify whether corneal densitometry can significantly decrease after lens discontinuation.

The present study has several limitations, such as small sample size and the retrospective design. However, it reported the changes of corneal densitometry after long-term orthokeratology treatment and the recovery after discontinuation for the first time. The results are still significant. In the future, it is necessary to conduct large sample prospective studies and incorporate additional corneal diagnostic instruments such as confocal microscopy to enrich the knowledge of corneal transparency after orthokeratology treatment.

In conclusion, corneal densitometry over the 0–10 mm diameter area increased significantly after long-term orthokeratology treatment for myopia, starting from the optical center at three months and gradually expanding outward. The fitting mode used for orthokeratology treatment affected corneal densitometry within the first year. Corneal densitometry can be used as an index to quantify corneal transparency after orthokeratology treatment.
